# Abnormalities in Gut Microbiota and Metabolism in Patients With Chronic Spontaneous Urticaria

**DOI:** 10.3389/fimmu.2021.691304

**Published:** 2021-10-15

**Authors:** Xin Wang, Wanyu Yi, Liting He, Shuaihantian Luo, Jiaqi Wang, Li Jiang, Hai Long, Ming Zhao, Qianjin Lu

**Affiliations:** ^1^ Department of Dermatology, Hunan Key Laboratory of Medical Epigenomics, The Second Xiangya Hospital of Central South University, Changsha, China; ^2^ Institute of Dermatology, Chinese Academy of Medical Sciences and Peking Union Medical College, Nanjing, China

**Keywords:** chronic spontaneous urticaria, feces, microbiota, metabolomic, pathogenesis, allergy, autoimmune disease

## Abstract

**Background:**

Increasing evidence suggests that the gut microbiome plays a role in the pathogenesis of allergy and autoimmunity. The association between abnormalities in the gut microbiota and chronic spontaneous urticaria (CSU) remains largely undefined.

**Methods:**

Fecal samples were obtained from 39 patients with CSU and 40 healthy controls (HCs). 16S ribosomal RNA (rRNA) gene sequencing (39 patients with CSU and 40 HCs) and untargeted metabolomics (12 patients with CSU and 12 HCs) were performed to analyze the compositional and metabolic alterations of the gut microbiome in CSU patients and HCs.

**Results:**

The 16S rRNA gene sequencing results showed a significant difference in the β-diversity of the gut microbiota, presented as the Jaccard distance, between CSU patients and HCs. No significant differences were found in the α-diversity of the gut microbiota between patients and HCs. At the phylum level, the major bacteria in the gut microbiome of patients with CSU were Firmicutes, Bacteroidetes, Proteobacteria, and Actinobacteria. At the genus level, *Lactobacillus*, *Turicibacter*, and *Lachnobacterium* were significantly increased and *Phascolarctobacterium* was decreased in patients with CSU. PICRUSt and correlation analysis indicated that *Lactobacillus*, *Turicibacter*, and *Phascolarctobacterium* were positively related to G protein-coupled receptors. Metabolomic analysis showed that α-mangostin and glycyrrhizic acid were upregulated and that 3-indolepropionic acid, xanthine, and isobutyric acid were downregulated in patients with CSU. Correlation analysis between the intestinal microbiota and metabolites suggested that there was a positive correlation between *Lachnobacterium* and α-mangostin.

**Conclusions:**

This study suggests that disturbances in the gut microbiome composition and metabolites and their crosstalk or interaction may participate in the pathogenesis of CSU.

## 1 Introduction

Chronic urticaria (CU), affecting 0.5%–1% of the general population, is defined as daily or an almost daily appearance of urticaria symptoms with or without angioedema. Approximately 80% of CU patients have no triggering physical stimuli or specific allergens identified, which is termed chronic spontaneous urticaria (CSU). CSU significantly affects patients’ performance at work and school and impairs quality of life. However, the etiology and pathogenesis of CSU are sophisticated and remain largely unclear.

The abnormal activation of mast cells and basophils is the basic pathogenic process in the onset of wheals and pruritus in CSU (1). A variety of potential mechanisms that trigger this final process in CSU pathogenesis have been proposed, among which the autoimmune nature of CSU, at least in a large proportion of CSU patients, has been supported by an increasing body of evidence (2). Research advances in recent decades have linked the alterations of the gut microbiota to the pathogenesis of autoimmune diseases such as lupus erythematosus (3) and that of allergic disorders such as food allergy (4), atopic dermatitis (5), and asthma (6). Between 2017 and 2018, two studies initially detected the abundance of several microbes, i.e., *Lactobacillus*, *Bifidobacterium*, *Bacteroides*, *Akkermansia muciniphila*, *Clostridium leptum*, *Faecalibacterium prausnitzii*, and Enterobacteriaceae, in fecal samples from patients with CU using real-time quantitative polymerase chain reaction (qPCR), which identified a relative decrease of *Lactobacillus*, *Bifidobacterium*, *A. muciniphila*, *C. leptum*, and *F. prausnitzii* in patients with CSU (7, 8). However, our understanding of the gut microbiome of patients with CSU is still quite limited.

With the development of experimental technologies, 16S ribosomal RNA (rRNA) gene sequencing has been applied as a standard method to illustrate a more comprehensive picture of the intestinal microbiome. Since 2019, several studies have begun to report alterations in the gut microbiome of patients with CSU using this method; however, the research findings were not consistent with each other and were limited by their small sample sizes (9, 10). In 2020, a combinational analysis incorporating gut microbiome data and serum metabolome data revealed a potential role of gut microbiota-associated alterations in unsaturated fatty acids and the butanoate metabolism pathway in the pathogenesis of CSU (11). This study has largely extended our knowledge of the potential impact of the gut microbiota on serum metabolism in CSU, which prompted us to ask the unanswered question of whether the metabolome in the gut microenvironment was also disturbed in patients with CSU and the potential association between the altered gut microbiome and gut metabolomics in the pathogenesis.

In this study, we performed 16S rRNA gene sequencing and untargeted metabolomics in fecal samples to examine the differences in the gut microbiome and metabolites between patients with CSU and healthy controls (HCs), with the aim to providing preliminary data for further investigation into the potential role of gut microbiota perturbance in the pathogenesis of CSU.

## 2 Materials and Methods

### 2.1 Study Population and Subject Matching

Thirty-nine patients with CSU and 40 healthy volunteers (12–60 years of age) were recruited from The Second Xiangya Hospital of Central South University between February 1 and October 31, 2018. The diagnosis of CSU was established according to the EAACI/GA²LEN/EDF/WAO guideline (12). None of the subjects had been exposed to treatment with antibiotics or probiotics during the past 3 months prior to the collection of fecal samples. Patients were excluded if they had severe systemic diseases affecting vital organs, immunodeficiency disorders, auto-inflammatory syndromes, urticarial vasculitis, severe infections, or inducible urticaria except for dermographism or if they had been treated with systemic corticosteroids, biologics, or immunosuppressants in the past 3 months. Healthy volunteers without a personal history or family history of urticaria, atopic dermatitis, allergic rhinitis, conjunctivitis, asthma, anaphylaxis, autoimmune diseases, or other severe systemic diseases were recruited into the HCs group. This study was approved by the Medical Ethics Committee of The Second Xiangya Hospital of Central South University. Written informed consent was obtained for each participant before sample collection.

### 2.2 Sample Collection

Subjects provided fresh feces within 1 day after enrollment. Feces were collected using Longseegen stool storage kit and suspended in stool storage solution according to the manufacturer’s protocol (Longsee Biomedical, Guangzhou, China) and stored at 4°C before further analysis.

### 2.3 DNA Extraction and 16S rRNA Gene Sequencing

The gut microbiota of the subjects was determined with 16S rRNA gene sequencing analysis, as described previously (13). 16S rRNA gene sequencing was performed at Longsee Biomedical Corporation (Guangdong, China). Briefly, the microbial DNA was extracted from the fecal sample from each participant using LONGSEE Stool DNA Kit (Longsee Biomedical, Guangzhou, China) in accordance with the manufacturer’s instructions. The forward primer (5′-ACT CCT ACG GGA GGCAGC AG-3′) and reverse primer (5′-GGA CTA CHV GGG TWT CTA AT-3′) were used to amplify the 16S rRNA gene V3–V4 variable region from the bacterial DNA by PCR. Amplifications were performed using a step cycling protocol consisting of 95°C for 3 min, 30 cycles of denaturation at 95°C for 15 s, annealing at 55°C for 15 s, and extension at 72°C for 30 s, followed by a final extension period at 72°C for 5 min. The PCR-amplified product was purified, quantified, normalized, and sequenced on the Illumina MiSeq-PE250 instrument using a Miseq Reagent Kit V3 (MS-102-3003; Illumina, San Diego, CA, USA). Raw FastQ files were demultiplexed, quality-filtered by Trimmomatic, and merged using FLASH (Fast Length Adjustment of Short Reads) (14). Trimmed sequences were clustered to operational taxonomic units (OTUs) with a 100% similarity cutoff using QIIME2 (Quantitative Insights Into Microbial Ecology, version 2) (15), and chimeric sequences were identified and removed using UCHIME, an algorithm detecting chimeric sequences with two or more segments (16). The taxonomical assignment of the OTUs was performed with QIIME2 classify-sklearn against the Silva database (http://www.arb-silva.de/) using a confidence threshold of 97%.

### 2.4 Metabolite Extraction and UHPLC-MS/MS Analysis

Stool samples were centrifuged at 17,000 × *g* for 15 min at 4°C. The supernatant was transferred to a new 1.5-ml centrifuge tube and dried by vacuum. Then, 200 μl of a 50% acetonitrile aqueous solution was added, vortexed for 30 s, sonicated for 10 min, centrifuged at 17,000 × *g* for 15 min, and then filtered through a 0.22-μm filter. Quality control (QC) samples were prepared by mixing aliquots of the supernatant from all samples. Liquid chromatography–tandem mass spectrometry (LC-MS/MS) analysis was performed using an ultra-high-performance liquid chromatography (UHPLC) system (1290 Infinity II LC System, Agilent Technologies, Santa Clara, CA, USA) and an ultra-performance liquid chromatography (UPLC) high-strength silica (HSS) T3 column (2.1 mm × 100 mm, 1.8 μm) coupled to a quadrupole time-of-flight (Q-TOF) mass spectrometer (6545 LC/Q-TOF MS, Agilent Technologies). Mobile phase A was 0.1% formic acid in water for positive mode and 0.5 mmol/L ammonium fluoride in water for negative mode; mobile phase B was acetonitrile. The elution gradient is set to: 0–1.0 min, 5% B; 1.0–2.0 min, 5%–10% B; 2.0–10.0 min, 10%–60% B; 10.0–14.0 min, 60%–95% B; 14.0–19.0 minutes, 95% B; 19.0–20.0 min, 95%–5% B; 20.0–23.0 min, 5% B. The flow rate was 0.4 ml/min. The injection volume was 1 μl. The Q-TOF mass spectrometer was used for its ability to acquire the MS/MS spectra on auto MS/MS mode in the control of the acquisition software (LC/MS Data Acquisition, version B.08.00, Agilent Technologies). The electrospray ionization (ESI) source conditions were set as follows: sheath gas flow rate, 12 L/min; sheath gas temperature, 350°C; dry gas flow rate, 8 L/min; dry gas temperature, 320°C; full MS resolution, 40,000; MS/MS resolution, 40,000; and collision energy, 20/40. In the normalized collision energy (NCE) mode, the spray voltage was 4.0 kV (positive) or −3.5 kV (negative). Mass Spectrometry–Data Independent Analysis software (MS-DIAL) was used for peak search, peak alignment, and other data processing, and the given identification results were obtained based on database matching with the first- and second-level maps.

### 2.5 Bioinformatics Analysis

For 16S rRNA gene sequencing analysis, diversity was calculated using the QIIME2 tool (15). The species richness and evenness within the bacterial populations were calculated by the α-diversity (17), whose metrics include Chao 1, evenness, Faith’s phylogenetic diversity (PD), Observed OTUs, and the Shannon and Simpson indices. The significance of α-diversity was calculated using Wilcoxon matched-pairs signed-rank test. The heterogeneity of the microbial communities, β-diversity, was determined using Jaccard distance matrices calculated by QIIME script, whose significance was also determined by PerMANOVA (permutational multivariate analysis of variance) (18). The greater the Jaccard distance, the less similarity among the microbial communities. Principal coordinates analysis (PCoA) was performed to visually present the individual and/or group differences in microbial distribution (19). Linear discriminant analysis (LDA) effect size (LEfSe) was performed to present the statistically significant differences in the relative abundance of gut microbial families and genera between two groups of samples (20). Only LDA values >2.5 and *p*-value <0.05 were considered significantly enriched. The performance of relevant microbes was measured by the area under the receiver operating characteristic (ROC) curve (AUC), which was computed by SPSS.

To evaluate differences in the predicted metabolic functions of the gut microbiota between CSU patients and HCs, Phylogenetic Investigation of Communities by Reconstruction of Unobserved States (PICRUSt) was performed to infer the metagenome functional content in the samples (21). Differences and changes in the metabolic pathways of functional genes of the microbial communities between CSU patients and HCs can be predicted by differential analysis of KEGG (Kyoto Encyclopedia of Genes and Genomes) metabolic pathways.

For metabolomics analysis, all putative identities were confirmed by matching with entries in the MassBank of North America (MoNA) (http://mona.fiehnlab.ucdavis.edu/), the HMDB database (http://www.hmdb.ca), and the KEGG database (http://www.genome.jp/kegg) using the molecular mass data (*m*/*z*) of samples. The metabolite would be identified when a mass difference between the observed value and the database value was <0.025 Da. The datasets were analyzed using MetaboAnalyst 3.0 in a pattern recognition method. Peak areas were normalized to internal standards, as previously described in detail (22). Metabolites with a percentage relative SD of >30% in the quality control samples were excluded, and the remaining data were log transformed. For univariate analysis, the statistical significance of features between CSU patients and HCs was determined by *t*-test using MetaboAnalyst 3.0. For multivariate statistical analysis, partial least squares discrimination analysis (PLS-DA) was carried out on mean-centered and unit variance-scaled data. Metabolites were ranked according to their variable importance in the projection (VIP) scores from the PLS-DA model. Metabolites with VIP scores >1.0 in the multivariate statistical analysis and *p* < 0.05 in the univariate analysis were considered as the significant contributors. A metabolomics pathway analysis was performed using MetaboAnalyst 3.0 to determine the most relevant metabolic pathways involved in CSU. We assessed a test both for overrepresentation of altered metabolites within a pathway (hypergeometric tests) (23) and for the impact of the altered metabolites on the pathway functions through changes in critical junction points for the pathway (relative betweenness centrality) (24). The most significant pathways, in terms of hypergeometric test *p*-value and impact, were shown by plotting the results of KEGG pathways. GraphPad Prism 7.0 was used to obtain Spearman’s correlation coefficients between metabolites and intestinal microbiota.

### 2.6 Statistical Analysis

Data in a normal distribution was expressed as mean (±SD). Percentage is used for enumeration data. Student’s *t*-test or Mann–Whitney *U* test was performed to test the differences of two groups. Statistical analyses were performed using SPSS Statistics (v.26.0.0.0; SPSS Inc., Chicago, IL, USA), GraphPad Prism software (version 7.0, GraphPad Software, San Diego, CA, USA), or R software (version 3.4.4). *P*-values <0.05 were considered significant.

### 2.7 Data Availability

The 16S sequencing data of this study have been deposited in the SRA accession database (ID: PRJNA721617).

## 3 Results

### 3.1 General Characteristics of Participants

In this study, we enrolled 79 subjects, including 39 patients with CSU and 40 HCs. No significant differences were found in age, sex ratio, and body mass index (BMI) between the two groups (*p* > 0.05). The baseline characteristics of the subjects, including a series of clinical features of the patients with CSU, are provided in **Table 1**.

**Table 1:** Demographic and clinical characteristics of participants.

Features	CSU patients (*n* = 39)	HCs (*n* = 40)	*p*-value
Age (years), mean ± SD	38.8 ± 10.9	38.8 ± 10.5	0.98
Sex: female, *n* (%)	23 (59)	60	0.93
BMI (kg/m^2^), mean ± SD	23.9 ± 3.1	22.6 ± 3.7	0.09
Time since diagnosis of CSU (months), median (IQR)	12 (5–48)	–	–
Age of onset CSU (years), mean ± SD	35.6 ± 11.2	–	–
Presence of angioedema, *n* (%)	7 (17.9)		
Concomitant symptom, *n* (%)			
Nausea and vomiting	3 (7.7)	–	–
Chills and fever	3 (7.7)	–	–
Headache and dizziness	2 (5.1)	–	–
Abdominal pain and diarrhea	1 (2.6)	–	–
Arthralgia	1 (2.6)	–	–
Fatigue	14 (35.9)	–	–
Syncope	1 (2.6)	–	–
Dermographism, *n* (%)	22 (56.4)	–	–
Contagious disease, *n* (%)			
Hepatitis B	5 (12.8)	–	–
Poor sleep quality, *n* (%)	20 (51.3)	–	–
Poor appetite, *n* (%)	4 (7.7)	–	–
Unformed stool, *n* (%)	3 (7.7)	–	–
Education level, *n* (%)			
Elementary school	7 (17.9)	–	–
Middle school	13 (33.3)	–	–
High school	5 (12.8)	–	–
College and above	14 (35.9)	–	–
History of food allergy, *n* (%)	23 (59.0)	1 (2.5)	–
History of drug allergy, *n* (%)	9 (23.1)	1 (2.5)	–
Family history of urticaria, *n* (%)	7 (17.9)	–	–
Smoking, *n* (%)			
Current or former	15 (38.5)	–	–
Never/almost never	24 (61.5)	–	–
Drinking, *n* (%)			
Current or former	9 (23.1)	–	–
Never/almost never	30 (76.9)	–	–

CSU, chronic spontaneous urticaria; HCs, healthy controls; BMI, body mass index; IQR, interquartile range.

### 3.2 Gut Microbiome of Patients with CSU and HCs Based on 16S rRNA Gene Sequencing Data

#### 3.2.1 Comparison of Gut Microbiome Diversities Between Patients With CSU and HCs

**Figure 1:**
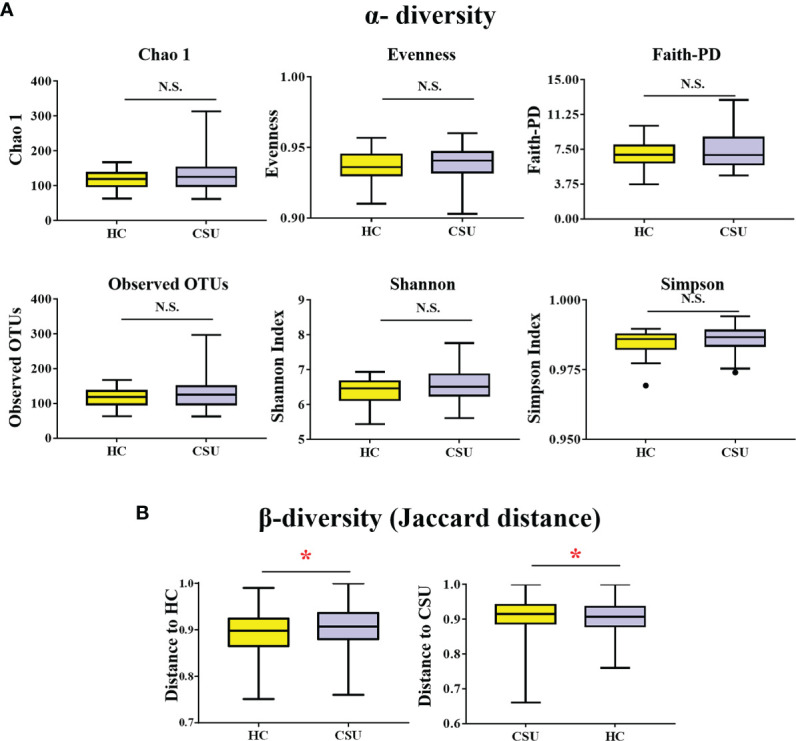
Comparing the diversity of the gut microbiota of patients with chronic spontaneous urticaria (CSU) and healthy controls (HCs). **(A)** The α-diversity of CSU patients and HCs was measured with the Chao 1 index, evenness, Faith’s phylogenetic diversity (PD), observed operational taxonomic units (OTUs), Shannon index, and Simpson index. There was no difference in α-diversity between the two groups (*p* > 0.05). **(B)** Group differences in β-diversity (Jaccard distance index) with respect to CSU patients and HCs. Statistical analyses were performed using PerMANOVA (*p < 0.05). N.S., not significant.

A total of 519,595 high-quality sequences (6,577 sequences per sample) were obtained from the fecal samples of 79 subjects (39 CSU patients and 40 HCs). To evaluate alterations in the gut microbial diversities between CSU patients and HCs, we examined the α-diversity using six indices, namely, Chao 1, evenness, Faith’s PD, observed OTUs, and the Shannon and Simpson indices (**Figure 1A** and **Supplementary Table S1**). No significant difference in the α-diversity of the gut microbiome between two groups was found (*p* > 0.05). For β-diversity (**Figure 1B** and **Supplementary Table S2**), we observed significant differences in the Jaccard distance between CSU patients and HCs (*p < 0.05).

#### 3.2.2 The Abundance of Intestinal Microbiota Taxa in Patients With CSU and HCs

**Figure 2:**
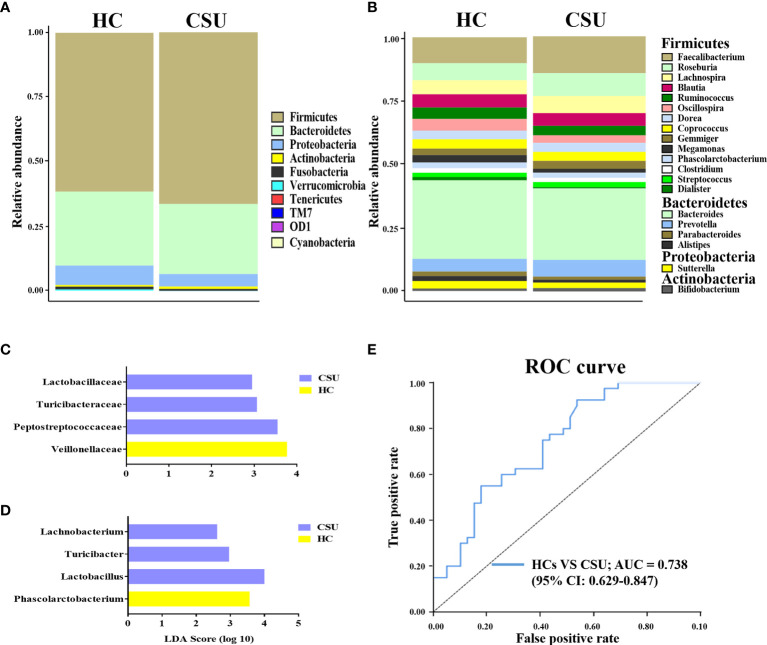
Abundance of fecal bacterial community between patients with chronic spontaneous urticaria (CSU) and healthy controls (HCs). **(A, B)** Profiles of the relative abundance at the phylum **(A)** and genus **(B)** levels between the two groups. Only the top 10 phyla and the top 20 genera are shown. **(C, D)** Linear discriminant analysis (LDA) demonstrated distinct gut bacterial taxa enriched in patients with CSU compared to HCs. (LDA score > 2, *p* < 0.05). Bacterial taxa that were enriched in fecal samples from patients with CSU (*blue*) and HCs (*yellow*) are shown by the LDA scores at the family **(C)** and genus **(D)** levels. **(E)** Receiver operating characteristic (ROC) curve of the combinations of selected families and genera to identify CSU patients and HCs. The area under the ROC curve (AUC) is shown for the microbial combination predictors with 95% CI.

The bacterial communities and relative abundance in the two groups were explored at different taxonomic levels. At the phylum level, the major phyla in the gut microbiota of patients with CSU were similar to that of HCs: Firmicutes, Bacteroidetes, Proteobacteria, and Actinobacteria (**Figure 2A** and **Supplementary Table S3**). The relative abundance of Firmicutes was increased in patients with CSU, while the relative abundance of Proteobacteria was decreased. At the genus level, we observed that the relative abundance of *Faecalibacterium*, *Roseburia*, *Lachnospira*, *Gemmiger*, *Prevotella*, and *Bifidobacterium* was increased in patients with CSU, whereas the relative abundance of *Blautia*, *Ruminococcus*, *Oscillospira*, *Megamonas*, *Dialister*, *Bacteroides*, *Parabacteroides*, *Alistipes*, and *Sutterella* was decreased in this group compared to HCs (**Figure 2B** and **Supplementary Table S4**).

To investigate the features more likely to explain the differences between CSU patients and HCs, LEfSe was performed (**Supplementary Table S5**). The log_10_ LDA scores were presented as a bar plot. In terms of the family level, LEfSe analysis identified Lactobacillaceae, Turicibacteraceae, and Peptostreptococcaceae as significantly more abundant and Veillonellaceae as less abundant in feces from patients with CSU compared to HCs (**Figure 2C**). At the genus level, the results demonstrated that *Lachnobacterium*, *Turicibacter*, and *Lactobacillus* were significantly enriched and *Phascolarctobacterium* was decreased in patients with CSU compared to HCs (**Figure 2D**). Taken together, these findings demonstrated that there was biased constitution in the gut microbial community of patients with CSU.

Given that the intestinal microbiome of patients with CSU was different from that of HCs, it would be interesting to figure out whether the intestinal microbiota could serve as potential biomarkers for CSU diagnosis and prediction. For this purpose, we adopted the relative abundances of the selected candidate families and genera mentioned above as predictors of CSU. ROC curve analysis was used to calculate the AUC when the intestinal microbiome was compared between CSU patients and HCs. The intestinal microbiome of patients with CSU was characterized by the increased relative abundance of Lactobacillaceae, Turicibacteraceae, Peptostreptococcaceae, *Lachnobacterium*, *Turicibacter*, and *Lactobacillus* and the decreased relative abundance of Veillonellaceae and *Phascolarctobacterium*. ROC analysis showed that the AUC values of each of these bacteria were 0.411 [95% confidence interval (CI) = 0.285–0.537], 0.422 (95% CI = 0.295–0.549), 0.376 (95% CI = 0.251–0.5), 0.448 (95% CI = 0.321–0.576), 0.437 (95% CI = 0.31–0.564), 0.424 (95% CI = 0.298–0.551), 0.663 (95% CI = 0.539–0.788), and 0.616 (95% CI = 0.49–0.741), respectively. We then attempted to combine these bacteria for better surveillance values in order to distinguish CSU patients from HCs. The AUC of the combination of Lactobacillaceae, Turicibacteraceae, Peptostreptococcaceae, *Lachnobacterium*, *Turicibacter*, *Lactobacillus*, Veillonellaceae, and *Phascolarctobacterium* was 0.738 (95% CI = 0.629–0.847) (**Figure 2E**). These results suggested that a disordered intestinal microbiome may have the potential to discriminate between CSU patients and HCs.

Among these eight altered families or genera, we further analyzed the potential correlation between the relative abundance of each gut microbe and clinical features such as BMI, time since diagnosis of CSU, complication with angioedema, history of food allergy, history of drug allergy, and family history of urticaria using clinical data of the 39 patients with CSU. Nonetheless, no correlation between the abundance of any of these eight gut microbes and any of the six clinical features was identified.

We also performed an analysis in which patients with CSU were divided into group A and group B based on their different patterns of gut microbial diversity (**Supplementary Figures S1A, B**). When we compared the clinical differences of patients in groups A and B, no significant difference in BMI, time since diagnosis of CSU, complication with angioedema, history of food allergy, history of drug allergy, or family history of urticaria was identified between the two subgroups of patients (**Supplementary Figures S1C–H**).

#### 3.2.3 Predicted Metabolic Functions of Characteristic Gut Microbiota in Patients With CSU

**Figure 3:**
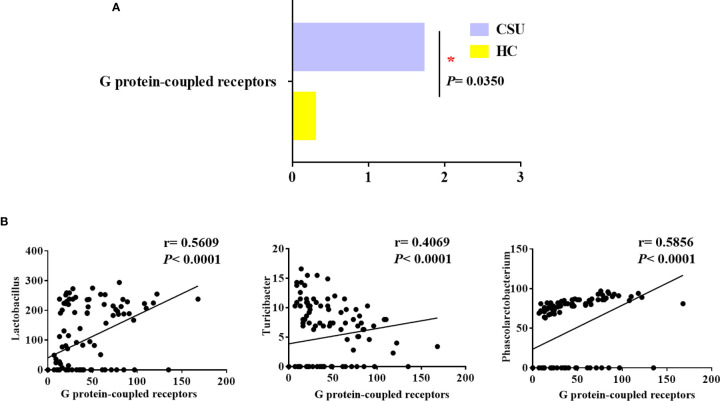
Distinct predicted metabolic pathways between patients with chronic spontaneous urticaria (CSU) and healthy controls (HCs). **(A)** The significantly different predicted metabolic pathways between CSU patients and HCs are shown by the Mann–Whitney test (*p < 0.05). **(B)** Association of the abundance of *Lactobacillus*, *Turicibacter*, and *Phascolarctobacterium* with G protein-coupled receptors. (Spearman’s rank correlation coefficient, *r* values, and *p*-values are shown).

To evaluate functional differences in the gut microbiomes of CSU patients *versus* HCs, we performed PICRUSt (21) to calculate their abundance, assign them to metabolic pathways using KEGG (25), and then test the differences between two groups. Compared with HCs, the G protein-coupled receptor (GPCR) pathway was the only enriched metabolic pathway associated with the gut microbiome in the CSU group among the 328 metabolic pathways tested (**Figure 3A** and **Supplementary Table S6**).

We next examined whether these bacteria, namely, Lactobacillaceae, Turicibacteraceae, Peptostreptococcaceae, *Lachnobacterium*, *Turicibacter*, *Lactobacillus*, Veillonellaceae, and *Phascolarctobacterium*, which were mentioned above as predictors of CSU, were related to GPCRs in order to gain insights into how specific taxa act in the aberrant metabolic pathways of CSU. Interestingly, in the CSU group, *Lactobacillus*, *Turicibacter*, and *Phascolarctobacterium* were positively related to GPCRs (**Figure 3B**). This indicated that the altered gut microbiome of patients with CSU may contribute to the progression of CSU *via* its impact on GPCRs.

### 3.3 Analysis of Gut Metabolomic Differences Between Patients With CSU and HCs

#### 3.3.1 Gut Metabolomic Profiles

**Figure 4:**
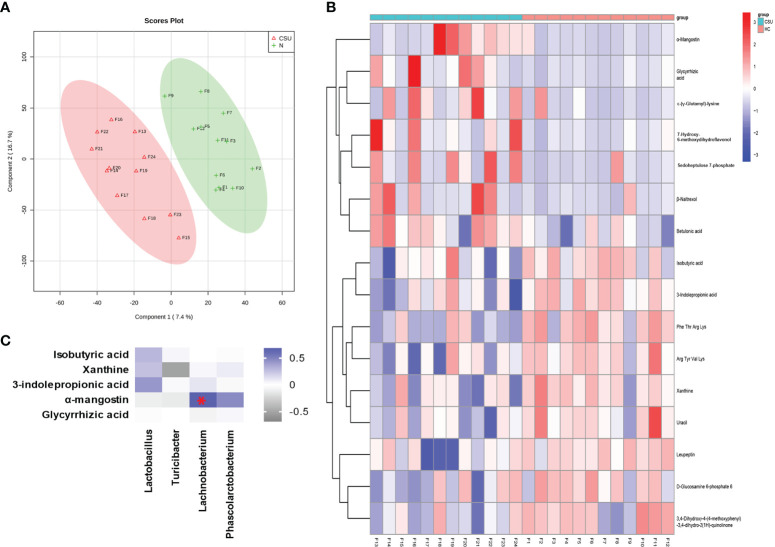
Identification of the metabolic signatures between patients with chronic spontaneous urticaria (CSU) and healthy controls (HCs). **(A)** Partial least squares discrimination analysis (PLS-DA) of fecal metabolomic data from CSU patients and HCs. The *red* and *green markers* represent CSU patients and HCs, respectively. **(B)** Fecal metabolic patterns in CSU patients and HCs shown as a heatmap. *Rows* represent data for metabolites and *columns* represent the subjects. *Red* and *blue colors* represent increased and decreased levels, respectively, of metabolites in patients with CSU compared to those in HCs. **(C)** Correlation for the significantly changed fecal metabolites and microbial genera. The square with a red asterisk refers to |*r*| > 0.60 and *p* < 0.05. *Blue* indicates a positive correlation while *white* indicates a negative correlation.

Metabolome refers to a collection of small-molecule compounds that are involved in the metabolism of organisms and maintain the normal growth and development of organisms. These are mainly referred to as the endogenous small molecules with relative molecular weight of less than 1,000. We performed metabonomic analysis of the metabolites extracted from 24 fecal samples (12 from the 39 patients with CSU and 12 from the 40 HCs). A total of 6,377 molecular features were obtained and subjected to statistical analysis using MetaAnalyst 3.0. PLS-DA revealed that CSU patients and HCs exhibited clear clustering with a *Q* ^2^ of −0.24 and a *R* ^2^ *Y* of 0.88, which indicated that the model was not overlifting and was reliable (**Figure 4A**). Features with VIP scores >1.0 in the multivariate statistical analysis and *p* < 0.05 in the univariate analysis were considered as significant differential metabolites and were visualized through heatmaps (**Figure 4B**). Five significantly differential metabolites with a second-level map were altered in the feces of patients with CSU compared with those of HCs (**Supplementary Table S7**). Among them, α-mangostin (α-MG) and glycyrrhizic acid were upregulated and 3-indolepropionic acid (IPA), xanthine, and isobutyric acid were downregulated in patients with CSU.

We then analyzed the correlation between the abundance of these five metabolites in fecal samples and clinical features such as BMI, time since diagnosis of CSU, complication with angioedema, history of food allergy, history of drug allergy, and family history of chronic urticaria using data of these 12 patients with CSU. Interestingly, the fecal abundance of xanthine was negatively correlated with the time since diagnosis of CSU in these patients (Spearman’s correlation analysis: *r* = −0.662, *p* = 0.0227) (**Supplementary Figure S2A**). Compared to the eight patients whose time since diagnosis of CSU is less than 12 months, the four patients with at least 12 months since diagnosis of CSU showed a significantly lower fecal abundance of xanthine (unpaired *t*-test: *p* = 0.0028) (**Supplementary Figure S2B**). Meanwhile, compared to the nine patients without a history of angioedema, the three patients complicated with angioedema showed a significantly higher fecal abundance of IPA (unpaired *t*-test: *p* = 0.0321) (**Supplementary Figure S2C**).

#### 3.3.2 Comparative Functional Differences of Metabolites in Patients With CSU and HCs

The significantly differential metabolites in the metabolic pathway were predicted using the KEGG pathway. Our results showed that some of the metabolic pathways in patients with CSU significantly changed compared with those of HCs, such as caffeine metabolism, protein digestion and absorption, and purine metabolism (**Supplementary Table S8**). The decreased xanthine was involved in caffeine metabolism and purine metabolism. The decreased isobutyric acid was involved in protein digestion and absorption.

### 3.4 Correlations Between Intestinal Microbiota and the Metabolites

Spearman’s correlation analysis was performed for the altered metabolites screened out in the fecal metabonomic analysis and the perturbed gut microbiota genera screened out by 16S rRNA gene sequencing analysis. The criteria for statistical significance were correlation coefficient |*r*| > 0.60 and *p* < 0.05. There was a positive correlation between *Lachnobacterium* and α-MG (**Supplementary Table S9**). A correlation heatmap was generated to present the covariation between the perturbed gut microbiota genera and the altered fecal metabolites, as shown in **Figure 4C**.

## 4 Discussion

In our study, we have profiled the intestinal microbiome and metabolites of patients with CSU using 16S rRNA gene sequencing and metabolomics analysis. Our major findings revealed that *Lactobacillus*, *Turicibacter*, and *Lachnobacterium* were significantly increased and *Phascolarctobacterium* was decreased in the CSU group, and the perturbed gut bacteria may contribute to the progression of CSU through GPCRs, suggesting that CSU is potentially associated with the disturbance of gut bacteria.

According to the β-diversity results, patients with CSU had distinct taxa compared with HCs, indicating that individual microbial communities were more different in patients with CSU, which was consistent with the findings of two recent studies on CSU (11, 26). A slight difference between our results and those of the recent study with a larger sample size was that we did not detect a significant difference in bacterial α-diversity compared with HCs, which may be partially due to differences in the sample size, the geographical background, dietary habits, and age between the subjects in the two studies (11). Nonetheless, researchers (27) found that microbial diversity measures did not differ between patients with food sensitization or food allergy and HCs. Therefore, the role of microbial diversity in allergy or CSU remains a subject of debate requiring further investigation. This also suggests that particular microbes might be more important than diversity in affecting the development of these diseases.

In the present study, the phyla Firmicutes, Bacteroidetes, Proteobacteria, and Actinobacteria were the most abundant microbes detected in all of the tested subjects. These results were consistent with those reported in previous studies (11, 28). At the genus level, LEfSe analysis of our data revealed that *Lactobacillus*, *Turicibacter*, and *Lachnobacterium* were significantly enriched in patients with CSU compared with HCs. Our data also showed that the abundance of eight families or genera, including these three bacteria, held the potential to discriminate patients with CSU from HCs according to the AUC analysis.

*Lactobacillus* spp. are Gram-positive facultative anaerobic rod-shaped bacteria capable of fermenting glucose and other sugars primarily to lactic acid or to lactic acid, carbon dioxide, and ethanol (29). *Lactobacillus* species maintain an acidic environment that limits the growth of other microorganisms and restores the physiological and microbial balance in human ecosystems (30). The role of *Lactobacillus* as a probiotic in the prevention and treatment of allergic diseases has been reported (31). Several studies reported that infants who have eczema or a family history of allergic disease have a lower abundance of Lactobacillaceae (genus *Lactobacillus*) (32, 33). However, the results of clinical trials and observational studies are inconsistent. *Lactobacillus* administration was ineffective in reducing the risk of eczema, asthma, or cow’s milk allergy (34, 35). The results in our study were consistent with the findings of a previous qPCR-based study that revealed a decrease in the relative amount of *Lactobacillus* in patients with CU (8). Interestingly, children with food sensitization exhibited significant increases in the numbers of *Lactobacillus* and Lactobacillaceae (36). Thus, intestinal abundance of *Lactobacillus* species and their function in the pathogenesis of CSU and allergy need further exploration.

*Turicibacter* are Gram-positive, anaerobic, non-spore-forming bacteria (37). Gastrointestinal *Turicibacter* species (family Turicibacteraceae) are reliant on host immune cells and bacterial sensors for survival. Studies have shown that two immunodeficient mouse models (innate immune deficient and B cell and T cell deficient) (38) and Toll-like receptor-2 knockout mice (39) had completely abolished *Turicibacter* within the gastrointestinal tract compared with wild-type mice, suggesting an interaction between these bacteria and host immune regulation. In 2019, Fung et al. (40) found that *Turicibacter sanguinis* expresses a neurotransmitter sodium symporter-related protein with sequence and structural homology to serotonin (5-hydroxytryptamine), which is partly responsible for the acute symptoms in allergic conditions such as allergen-induced asthma, urticaria, and anaphylaxis (41). Thus, the biological interaction between *Turicibacter* and CSU, which has not been previously reported, warrants further investigation.

*Lachnobacterium* spp., a member of the family Lachnospiraceae, are Gram-positive, obligately anaerobic, long straight rod-shaped bacteria that can ferment glucose to primarily lactic acid with very minor amounts of acetic and butyric acids (42). Shen et al. (43) found that, in the elderly group (≥60 years), age was negatively correlated with the presence of *Lachnobacterium*, and the aging-related decreases in *Lachnobacterium* among the tested participants could negatively affect their health (43). Another study (44) found that *Lachnobacterium* species were significantly decreased throughout infancy among children who developed atopic dermatitis and asthma. Our results showed that *Lachnobacterium* was abundant in the CSU group, but its biological significance needs to be further explored. According to the correlation analysis, *Lachnobacterium* species show a correlation with α-MG, which was abundant in the CSU group. α-MG, one of the major xanthone derivatives, exhibit a variety of effects, including anti-inflammatory, anti-microbial, anti-malarial, anticancer, anti-obesity, and cytotoxic effects. It also controls free radical oxidation and maintains the health of the cardiovascular and gastrointestinal systems (45). However, its function on allergic diseases is still equivocal.

Our correlation analysis suggested that *Lactobacillus*, *Turicibacter*, and *Phascolarctobacterium* may be correlated with the pathogenesis of CSU through GPCRs. GPCRs represent the largest family of seven-transmembrane domain receptors that regulate vital cellular functions such as cell proliferation, development, survival, metabolism, and neuronal signal transmission (46). Members of this family include GPR43, GPR41, GPR109A, GPR120, GPR40, GPR84, GPR35, GPR91, and GPR65 (47). Signaling through GPCRs generally has anti-inflammatory effects, and insufficient signaling through one or more GPCRs likely contributes to human diseases such as asthma, food allergies, and inflammatory bowel diseases (48). There is an increasing body of evidence showing that GPCRs are directly involved in mast cell (MC) degranulation and the release of inflammatory mediators (49). The various GPCRs expressed on MCs (50) may play an important role in human allergic diseases (50). Researchers found that human MCs are activated and secrete histamine in response to antimicrobial host defense peptides, neuropeptides, major basic protein, and eosinophil peroxidase mediated by the Mas-related GPCR X2 (MRGPRX2) (50, 51). Activation of the Mas-related GPCR X1 (MRGPRX1) by Der p1, a major allergen from house dust mite, may contribute to allergy and inflammation (52). GPR43 played a role in a mouse ovalbumin model of asthma (53). Short-chain fatty acid (SCFA) receptors (GPR41, GPR43, and GPR109A) and GPR84 were implicated in food allergy (54, 55). Prostaglandins (PGs) affected allergic inflammation *via* GPCRs (56). Thus, the GPCRs expressed on MCs may serve as potential drug targets for the treatment of allergic diseases (57). Researchers found that *Lactobacillus acidophilus* protected against the development of allergic inflammation by upregulating SCFAs and their receptors, GPR41/43 (58). The association between GPCRs and *Turicibacter* or *Phascolarctobacterium*, and how they participate in the pathogenesis of CSU, has not been reported.

Metabolomics enables a large-scale, qualitative, and quantitative study of metabolites in a systems biology approach. In this study, the gut metabolomic analysis showed that isobutyric acid, IPA, and xanthine were decreased in patients with CSU. Isobutyric acid is one of the short-chain fatty acids that can stimulate the proliferation of regulatory T cells (Tregs), a T-cell subset that plays an important role in inhibiting the development of allergic and autoimmune diseases (59). Moreover, the frequency of peripheral Tregs has been reported as significantly decreased in patients with CU (60). Thus, our data corroborate previous knowledge. IPA, a dietary tryptophan metabolite, is produced completely by the commensal gut bacteria *Clostridium sporogenes* (61) and absorbed from the gut into the blood stream (62) and cerebrospinal fluid (63). IPA exerts anti-inflammatory and antioxidative effects. Its pathogenic effect on angioedema is still unclear. Xanthine, a purine derivative, is produced during the degradation of adenosine triphosphate (ATP). Inhibition of inflammation is an important pharmacological action of xanthine (64). A state of inflammation is present in patients with CSU (65), which may be related to a decrease in the anti-inflammatory components, including, but not limited to, IPA and xanthine. Yu et al. (66) found that the altered metabolic activity of the purine cycle is also linked with allergic asthma (66). Furthermore, lower levels of xanthine in the cerebrospinal fluid are associated with depression, which may partly explain the high prevalence of depression in CSU patients (67). Our data also showed an interesting correlation between xanthine and the time since diagnosis of CSU, although it needs further validation in a larger sample size.

In summary, our data revealed disordered gut microbial constitution and metabolites using high-throughput molecular techniques, which provided potential clues for studying the pathogenesis of CSU. Our work establishes an important knowledge framework for further investigations into the molecular interactions between gut bacteria and metabolites and immunity, which would enhance our understanding of the mechanisms underlying the development of CSU. This progress may also provide novel potential targets for intervention of this sophisticated disease. However, a weak point of this study is that potential confounding factors such as age and diet were not taken into account during the design of this study. Due to the limitation of the methodology, fungi colonization was not assessed either. In addition, no discussion was performed on the differential metabolite glycyrrhizic acid, a chemical extracted from the root of the licorice plant, due to the lack of relevant material for detailed discussion. As a single-center study with a limited sample size, the generalizability of our findings should be cautiously appraised. Therefore, further multicenter studies with a larger sample size and more critical factors incorporated are warranted in future investigations.

## Data Availability Statement

The 16S rRNA data have been uploaded to NCBI: PRJNA721617.

## Ethics Statement

The studies involving human participants were reviewed and approved by the Medical Ethics Committee of The Second Xiangya Hospital of Central South University. Written informed consent to participate in this study was provided by each participant or by the participant's legal guardian/next of kin.

## Author Contributions

XW and HL designed the study. XW, WY, LH, SL, JW, and LJ performed the study. HL and MZ supervised the study. XW wrote the paper. HL, MZ, and QL revised the manuscript. All authors contributed to the article and approved the submitted version.

## Funding

This work was supported by the National Natural Science Foundation of China (81773334 and 81703133), Hu-Xiang Talents Program (2018RS3031), and the Clinical Medicine Research Center Program (2016SK4001) of Science-Technology Department of Hunan Province and Outstanding Innovation Talents Program of Changsha Science-Technology Bureau (kq1802002).

## Conflict of Interest

The authors declare that the research was conducted in the absence of any commercial or financial relationships that could be construed as a potential conflict of interest.

## Publisher’s Note

All claims expressed in this article are solely those of the authors and do not necessarily represent those of their affiliated organizations, or those of the publisher, the editors and the reviewers. Any product that may be evaluated in this article, or claim that may be made by its manufacturer, is not guaranteed or endorsed by the publisher.

## Supplementary Material

Supplementary Figure 1 Comparison of clinical features between two subgroups of CSU patients with distinct patterns of gut microbiome. **(A)** Patients with CSU (shown as red dots) were divided into two subgroups, Group A and Group B, by an oblique line draw in the plots depicted by PcoA analysis. **(B)** Gut microbial families and genera with statistically significant differences in relative abundance between Group A and Group B of CSU patients, as calculated by the LefSe analyses. **(C‒H)** Comparisons of patients’ clinical features including BMI **(C)**, time since diagnosis of CSU **(D)**, complication with angioedema **(E)**, history of food allergy **(F)**, history of drug allergy **(G)**, and family history of urticaria **(H)**, between Group A and Group B.

Supplementary Figure 2 The correlation between the abundance of significant differential metabolites and clinical features of CSU patients. **(A)** Spearman correlation analysis was performed between xanthine and the time since diagnosis of CSU (r= -0.662, *P*= 0.0227). **(B)** Fecal abundance of xanthine between patients whose time since diagnosis of CSU is less than 12 months (N=8) and those who have at least 12 months since diagnosis of CSU (N=4) (*P* =0.0028, unpaired *t* test). **(C)** Fecal abundance of 3-indolepropionic acid between patients with a history of angioedema (N=9) and those without (*P* =0.0321, unpaired *t* test).

Supplementary Table 1 Comparison of α-diversities between patients with CSU and HCs.

Supplementary Table 2 Comparison of β-diversities between patients with CSU and HCs.

Supplementary Table 3 The relative abundance of bacterial communities at phylum level.

Supplementary Table 4 The relative abundance of bacterial communities at genus level.

Supplementary Table 5 Linear discriminant analysis (LDA) effect size (LEfSe) between patients with CSU and HCs.

Supplementary Table 6 Predicted metabolic functions of characteristic gut microbiota in patients with CSU.

Supplementary Table 7 Gut metabolomic profiles between patients with CSU and HCs.

Supplementary Table 8 Comparative functional differences of metabolites in patients with CSU and HCs.

Supplementary Table 9 Correlations between intestinal microbiota and the metabolites.
