# Supplementary figures and images for: Abnormalities in Gut Microbiota and Metabolism in Patients With Chronic Spontaneous Urticaria
Source: Front Immunol. 2021 Oct 15;12:691304. doi: 10.3389/fimmu.2021.691304 (PMC8554312; doi:10.3389/fimmu.2021.691304)

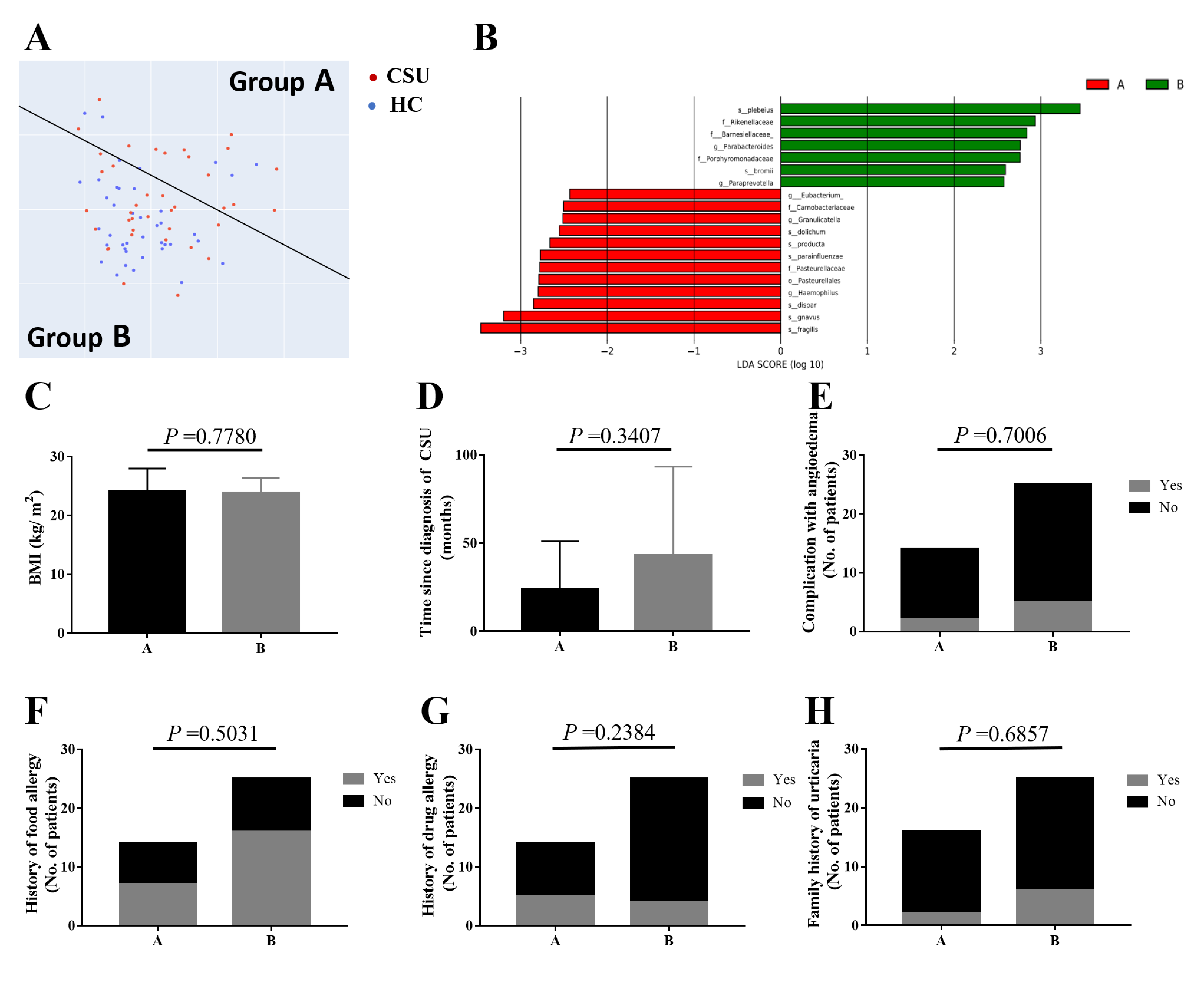

Supplement: Supplementary Figure 1 — Comparison of clinical features between two subgroups of CSU patients with distinct patterns of gut microbiome. (A) Patients with CSU (shown as red dots) were divided into two subgroups, Group A and Group B, by an oblique line draw in the plots depicted by PcoA analysis. (B) Gut microbial families and genera with statistically significant differences in relative abundance between Group A and Group B of CSU patients, as calculated by the LefSe analyses. (C‒H) Comparisons of patients’ clinical features including BMI (C), time since diagnosis of CSU (D), complication with angioedema (E), history of food allergy (F), history of drug allergy (G), and family history of urticaria (H), between Group A and Group B. [file Image_1.tif]

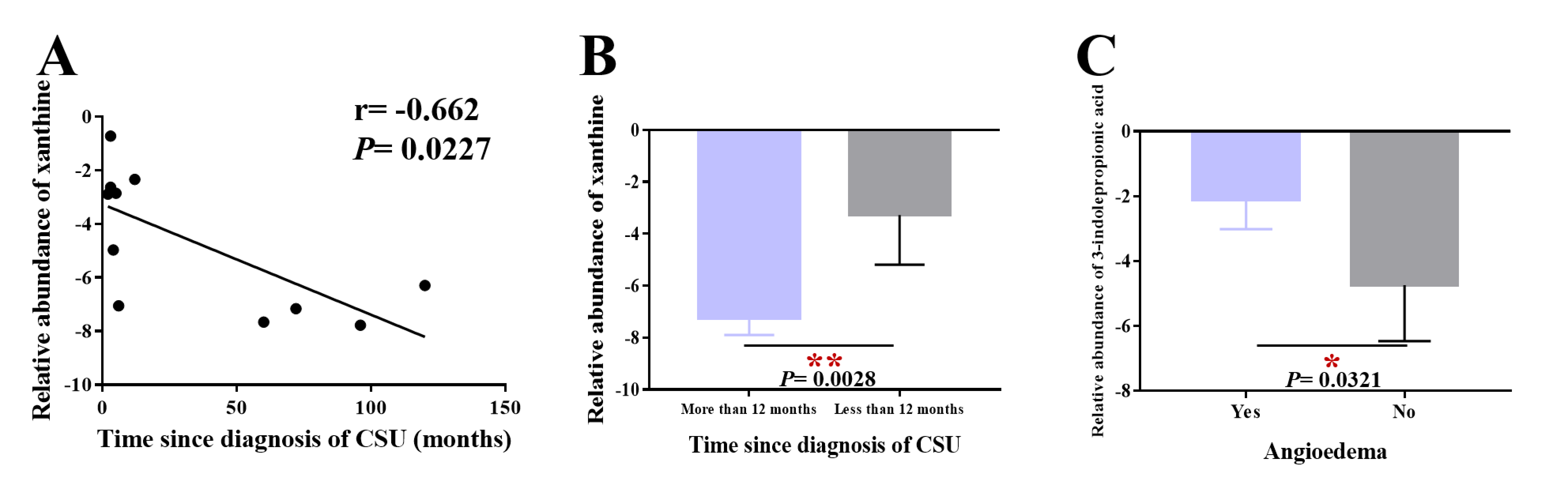

Supplement: Supplementary Figure 2 — The correlation between the abundance of significant differential metabolites and clinical features of CSU patients. (A) Spearman correlation analysis was performed between xanthine and the time since diagnosis of CSU (r= -0.662, P= 0.0227). (B) Fecal abundance of xanthine between patients whose time since diagnosis of CSU is less than 12 months (N=8) and those who have at least 12 months since diagnosis of CSU (N=4) (P =0.0028, unpaired t test). (C) Fecal abundance of 3-indolepropionic acid between patients with a history of angioedema (N=9) and those without (P =0.0321, unpaired t test). [file Image_2.tif]
